# EGCG-Functionalized Selenium Nanoparticles Mitigate High-Fat Diet-Induced Hepatic Lipotoxicity Through Keap1/Nrf2 Redox Modulation and Transcriptional Regulation of AMPK/SIRT1/PGC-1α/MFN2-Associated Mitochondrial Homeostasis

**DOI:** 10.3390/ijms27135768

**Published:** 2026-06-26

**Authors:** Fatma Al-Zahraa Sayed, Mennat allah Maher, Mariam Elsayed Elborlosy, Mennat Allah Safwat, Mariam Sayed Mahmoud, Fatma Y. Elmahdy, Romaysaa Tarek, Ahmed Hassan Ibrahim Faraag, Khaled Abuelhaded, Ahmed M. Ashour, Ali Khames, Khaled M. Alam-ElDein, Mohamed H. A. Gadelmawla

**Affiliations:** 1 School of Biotechnology, Badr University in Cairo (BUC), Badr City 11829, Egypt; 2 Botany and Microbiology Department, Faculty of Science, Helwan University, Ain Helwan 11795, Egypt; 3 Department of Pharmacology and Toxicology, College of Pharmacy, Umm Al-Qura University, P.O. Box 13578, Makkah 21955, Saudi Arabia; 4 Department of Pharmacology and Toxicology, Faculty of Pharmacy, Sohag University, Sohag 82511, Egypt; 5 Department of Life Sciences, Faculty of Biotechnology, Sinai University, Kantara Branch, Ismailia 41636, Egypt

**Keywords:** EGCG-functionalized selenium nanoparticles, HFD-induced hepatic lipotoxicity, Keap1/Nrf2 antioxidant axis, NF-κB-mediated inflammation, AMPK/SIRT1/PGC-1α/MFN2 signaling, mitochondrial apoptosis

## Abstract

High-fat diet (HFD)-induced hyperlipidemia is an experimental metabolic condition characterized primarily by dysregulated serum lipid levels and hepatic lipid accumulation, with associated oxidative, inflammatory, mitochondrial, and cardiovascular alterations. This study investigated the therapeutic efficacy of epigallocatechin gallate (EGCG)-functionalized selenium nanoparticles (EGCG-SeNPs) against HFD-induced metabolic and hepatic injury, in comparison with free EGCG, sodium selenite (Na_2_SeO_3_), and Lipanthyl. EGCG-SeNPs were characterized by dynamic light scattering, zeta potential analysis, transmission electron microscopy, X-ray diffraction, and UV–visible spectrophotometry. Forty-two adult male rats were allocated into six groups: control, HFD, HFD/Lipanthyl, HFD/EGCG, HFD/Na_2_SeO_3_, and HFD/EGCG-SeNPs. High-fat diet (HFD) feeding induced pronounced dyslipidemia, elevated hepatic enzymes, increased cardiac injury biomarkers, enhanced lipid peroxidation and nitrosative stress, depletion of antioxidant defenses, and disruption of the Kelch-like ECH-associated protein 1/nuclear factor erythroid 2-related factor 2 (Keap1/Nrf2) regulatory axis. HFD also increased nuclear factor-kappa B (NF-κB), tumor necrosis factor-alpha (TNF-α), and interleukin-6 (IL-6), while altering mitochondrial apoptotic markers, including B-cell lymphoma 2 (Bcl-2), cytochrome c, and caspase-3. At the transcriptional level, HFD increased lipogenic gene expression and reduced the expression of genes related to fatty-acid oxidation, metabolic regulation, and mitochondrial homeostasis. EGCG-SeNPs showed the greatest overall improvement among the tested interventions, as indicated by an improved lipid profile, hepato-cardiac injury biomarkers, antioxidant status, inflammatory markers, apoptotic markers, hepatic architecture, and Nrf2 immunoreactivity. Collectively, EGCG-SeNPs may mitigate HFD-induced hepatic lipotoxicity and associated cardiac stress through coordinated modulation of lipid metabolism, redox balance, inflammation, and mitochondrial homeostasis.

## 1. Introduction

**Figure 1:**
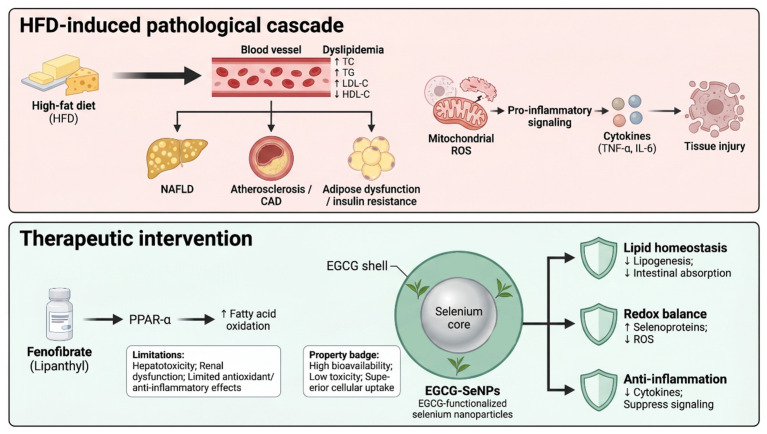
Pathological cascade of diet-induced hyperlipidemia and the synergistic rescue mechanism of EGCG-SeNPs. Prolonged intake of a high-fat diet disrupts lipid homeostasis, promoting systemic dyslipidemia, adipose tissue dysfunction, and excessive lipid deposition in vascular and hepatic tissues. At the molecular level, this lipid overload accelerates mitochondrial ROS overproduction, triggering a pro-inflammatory cytokine cascade and an apoptotic shift that together aggravate severe tissue injury. While conventional single-target therapies like fenofibrate exhibit clinical and physiological limitations, green-synthesized EGCG-SeNPs offer a potent, biocompatible intervention that restores lipid homeostasis through multiple targets, amplifies antioxidant defenses, and suppresses chronic metabolic inflammation. Created in BioRender. Ahms, A. BioRender.com/s96dv2v.

High-fat diet-induced hyperlipidemia is a prevalent metabolic disorder characterized by abnormal elevations in serum total cholesterol, triglycerides, and low-density lipoprotein cholesterol, accompanied by a reduction in high-density lipoprotein cholesterol. Prolonged intake of diets rich in saturated fats disrupts lipid homeostasis by enhancing hepatic lipogenesis, impairing lipid clearance, and promoting adipose tissue dysfunction. These alterations result in excessive lipid deposition in metabolic organs, particularly the liver and vascular system, thereby increasing susceptibility to metabolic syndrome and cardiovascular diseases. Consequently, high-fat diet-induced hyperlipidemia represents a major global health challenge with significant clinical and socioeconomic implications [1,2] (Figure 1).

The pathological consequences of hyperlipidemia extend well beyond dyslipidemia itself and include atherosclerosis, coronary artery disease, non-alcoholic fatty liver disease, and insulin resistance [3]. Epidemiological evidence consistently demonstrates a strong correlation between hyperlipidemia and increased cardiovascular morbidity and mortality, especially in populations exposed to unhealthy dietary patterns and sedentary lifestyles. At the molecular level, hyperlipidemia is closely associated with enhanced oxidative stress and chronic low-grade inflammation [4,5]. Excess circulating lipids stimulate the overproduction of reactive oxygen species and activate pro-inflammatory signaling cascades, leading to elevated levels of inflammatory cytokines and stress-related biomarkers that further aggravate tissue injury and metabolic dysfunction [6,7].

In recent years, natural products have attracted considerable attention as complementary or alternative therapeutic agents for the management of hyperlipidemia due to their multifaceted biological activities and favorable safety profiles [8,9,10]. Numerous phytochemicals exert lipid-lowering effects by regulating lipid metabolism, enhancing antioxidant defenses, and suppressing inflammatory responses. Among these, green tea is widely recognized for its health-promoting properties, which are largely attributed to its high content of polyphenolic catechins. Epigallocatechin gallate (EGCG), the most abundant and biologically active catechin in green tea, possesses a unique chemical structure rich in hydroxyl groups that confer potent antioxidant capacity [10,11]. EGCG has been reported to attenuate hyperlipidemia by inhibiting intestinal lipid absorption, suppressing hepatic lipid synthesis, and mitigating oxidative stress and inflammation [12,13].

Selenium is an essential trace element that plays a pivotal role in maintaining redox balance and regulating lipid metabolism through its incorporation into selenoproteins. These selenoproteins, including glutathione peroxidases and thioredoxin reductases, protect cellular components from oxidative damage and lipid peroxidation [14]. Selenium deficiency has been associated with dyslipidemia, enhanced oxidative stress, and inflammatory responses, whereas appropriate selenium supplementation has demonstrated beneficial effects on lipid profiles and inflammatory mediators. Therefore, selenium represents a critical micronutrient with potential therapeutic relevance in the prevention and management of hyperlipidemia [15].

Recent advances in nanotechnology have led to the development of selenium nanoparticles (SeNPs) as an innovative approach to improve selenium bioavailability and reduce its toxicity [16]. Compared with conventional selenium forms, SeNPs exhibit superior antioxidant activity, enhanced cellular uptake, and improved safety profiles. Experimental studies have shown that SeNPs effectively modulate lipid metabolism, suppress oxidative stress, and attenuate inflammatory signaling pathways in hyperlipidemic conditions. Moreover, biosynthesis of SeNPs using natural polyphenols may confer synergistic effects, further enhancing their protective efficacy against high-fat diet-induced metabolic disturbances [13].

Fenofibrate (Lipanthyl), a widely prescribed hypolipidemic drug, exerts its therapeutic effects primarily through activation of peroxisome proliferator-activated receptor-α, leading to enhanced fatty acid oxidation and reduced triglyceride levels [17]. Despite its clinical utility, fenofibrate therapy is associated with several limitations, including hepatotoxicity, renal dysfunction, and limited antioxidant and anti-inflammatory actions [18]. Therefore, there is a need to explore complementary multi-target strategies that can improve lipid metabolism while simultaneously modulating oxidative stress, inflammatory signaling, and mitochondrial dysfunction associated with HFD-induced hepatic injury.

The present study aimed to evaluate the therapeutic efficacy of epigallocatechin gallate-functionalized selenium nanoparticles (EGCG-SeNPs) in comparison with free EGCG, sodium selenite, and Lipanthyl in a rat model of high-fat diet-induced hyperlipidemia and hepatic injury. The study further sought to elucidate the molecular mechanisms underlying the potential protective effects of EGCG-SeNPs by assessing their ability to restore lipid homeostasis, attenuate hepatic oxidative stress and inflammation, regulate Keap1/Nrf2 antioxidant signaling, modulate lipogenesis and fatty-acid oxidation-related gene expression, preserve AMPK/SIRT1/PGC-1α/MFN2-associated mitochondrial homeostasis, and suppress cytochrome c/caspase-3-associated apoptotic injury.

## 2. Results

### 2.1. Characterization of EGCG-Loaded Selenium Nanoparticles

**Figure 2:**
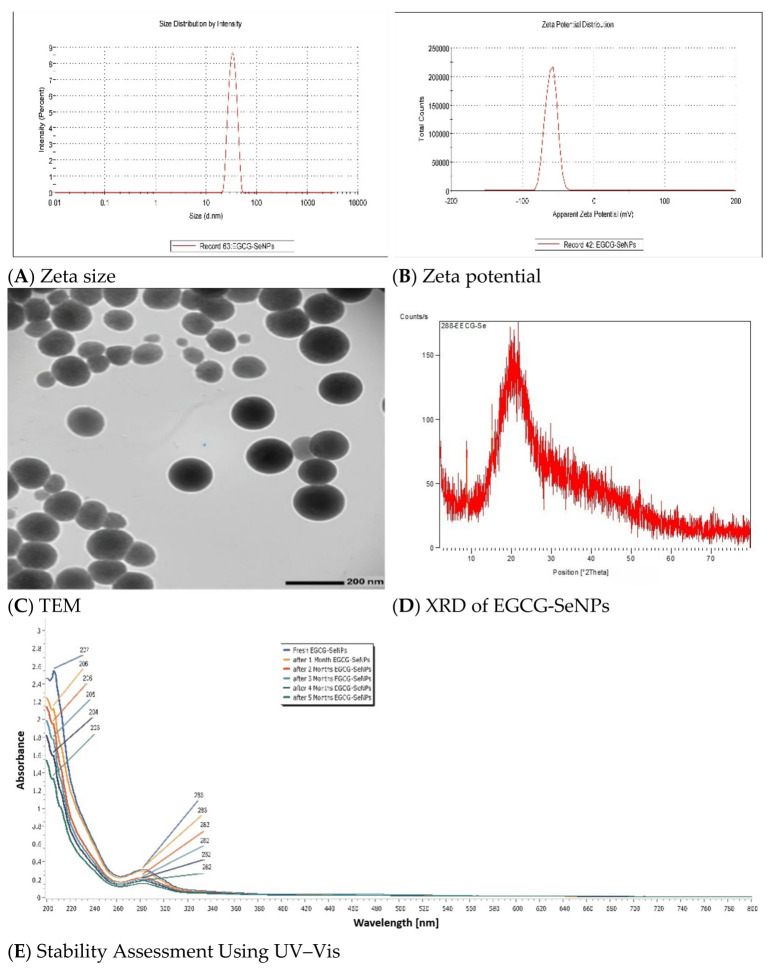
(**A**) Zeta size, (**B**) zeta potential, (**C**) TEM of selenium nanoparticles (scale bar = 200 nm), (**D**) XRD of EGCG-SeNPs, and (**E**) stability assessment using UV–Vis, showing successful preparation and good stability of EGCG-SeNPs.

Dynamic light scattering (DLS) analysis was performed to determine the hydrodynamic diameter and size distribution of the EGCG-SeNPs in suspension. The results showed a Z-average particle size of 55.2 nm, with a single dominant peak centered at 56.8 nm, accounting for 100% intensity (Figure 2A).

The surface charge and colloidal stability of the nanoparticles were evaluated by zeta potential analysis. The EGCG-SeNPs exhibited a strongly negative zeta potential of −63.4 mV, with a standard deviation of 6.76 mV. This high negative surface charge suggests strong electrostatic repulsion between particles, contributing to excellent dispersion stability and resistance to aggregation (Figure 2B).

The morphology and size of epigallocatechin gallate-functionalized selenium nanoparticles (EGCG-SeNPs) were characterized using transmission electron microscopy (TEM). TEM micrographs revealed that the synthesized EGCG-SeNPs were predominantly spherical in shape with a relatively uniform size distribution. The nanoparticles appeared well dispersed with minimal aggregation, indicating effective stabilization by EGCG. Based on the TEM images, the particle size was estimated to be in the nanometer range, consistent with nanoscale selenium particles (Figure 2C).

X-ray diffraction analysis of EGCG-SeNPs demonstrated distinct diffraction features, indicating the presence of structurally organized domains within the EGCG-associated selenium nanoparticle formulation. The observed diffraction profile supports successful structural modification following nanoparticle synthesis and suggests that EGCG contributed to the organization and stabilization of the selenium-based nanosystem (Figure 2D).

The UV–Vis spectra of EGCG-SeNPs recorded at baseline and during monthly follow-up showed preservation of the characteristic absorption profile throughout the investigated period. Fresh EGCG-SeNPs exhibited prominent absorption features at approximately 207 nm and 283 nm. During storage, the primary absorption maximum showed only a minor, gradual shift from 207 nm in the freshly prepared formulation to 203 nm after 5 months, whereas the second characteristic band remained nearly unchanged, shifting slightly from 283 nm to 282 nm. Although a progressive reduction in absorbance intensity was observed over time, no major displacement of the characteristic peaks or appearance of new spectral bands was detected (Figure 2E).

### 2.2. Investigation of Lipid Profile 

**Figure 3:**
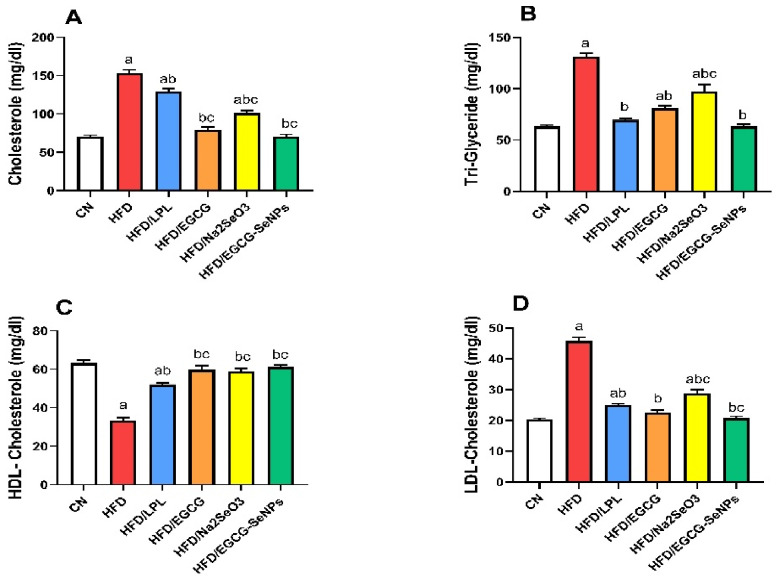
Effect of selenium, EGCG, and EGCG-SeNPs on lipid profile in high-fat diet-fed rats. (**A**) Total cholesterol, (**B**) triglycerides, (**C**) HDL-C, and (**D**) LDL-C levels in different experimental groups. Data are presented as mean ± SEM (n = 7). One-way ANOVA followed by Tukey’s post hoc test. a = vs. Control; b = vs. HFD; c = vs. HFD/LPL (*p* < 0.05).

As illustrated in Figure 3, HFD feeding significantly altered the serum lipid profile. Compared with the control group, total cholesterol increased from 70.41 ± 1.98 to 153.40 ± 4.19, triglycerides increased from 63.41 ± 1.46 to 131.20 ± 3.31, and LDL-C increased from 20.27 ± 0.47 to 45.83 ± 1.12, representing increases of 117.87%, 106.90%, and 126.06%, respectively. In contrast, HDL-C decreased from 63.22 ± 1.58 to 33.39 ± 1.56, corresponding to a 47.19% reduction relative to the control group.

Compared with the HFD group, Lipanthyl administration decreased total cholesterol, triglycerides, and LDL-C by 15.34%, 46.85%, and 45.55%, respectively, while HDL-C increased by 56.11%. EGCG treatment decreased total cholesterol, triglycerides, and LDL-C by 48.10%, 38.11%, and 50.81%, respectively, and increased HDL-C by 78.99% compared with HFD. Relative to the Lipanthyl group, epigallocatechin-3-gallate showed additional reductions in total cholesterol and LDL-C of 38.70% and 9.64%, respectively, and a 14.66% higher HDL-C level; however, triglycerides were numerically higher than in the Lipanthyl group (*p* < 0.05).

Sodium selenite treatment decreased total cholesterol, triglycerides, and LDL-C by 33.86%, 25.60%, and 37.13%, respectively, and increased HDL-C by 76.74% compared with HFD. Compared with Lipanthyl, sodium selenite showed lower total cholesterol by 21.88% and higher HDL-C by 13.21%, while triglycerides and LDL-C remained numerically higher than in the Lipanthyl group. The EGCG-SeNP group showed the largest numerical reductions in total cholesterol, triglycerides, and LDL-C compared with HFD, by 54.01%, 51.60%, and 54.68%, respectively, together with an 83.39% increase in HDL-C. Relative to Lipanthyl, EGCG-SeNPs showed reductions in total cholesterol, triglycerides, and LDL-C by 45.68%, 8.94%, and 16.76%, respectively, and an increase in HDL-C by 17.47%.

### 2.3. Liver Enzyme Test

**Figure 4:**
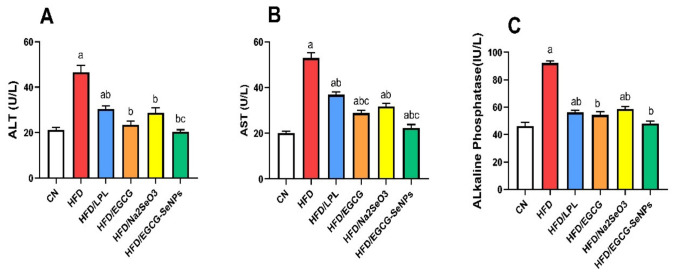
Effect of selenium, EGCG, and EGCG-SeNPs on liver function enzymes in high-fat diet-induced hepatic injury. (**A**) ALT, (**B**) AST, and (**C**) ALP serum activities in different groups. Data are presented as mean ± SEM (n = 7). One-way ANOVA followed by Tukey’s post hoc test. a = vs. Control; b = vs. HFD; c = vs. HFD/LPL (*p* < 0.05).

As shown in Figure 4, HFD feeding increased serum liver enzyme activities compared with the control group. ALT increased from 21.14 ± 1.26 to 46.53 ± 2.97, AST increased from 20.02 ± 0.82 to 52.93 ± 2.34, and ALP increased from 46.27 ± 2.54 to 92.18 ± 1.70, corresponding to increases of 120.14%, 164.45%, and 99.22%, respectively.

Compared with HFD, Lipanthyl decreased ALT, AST, and ALP by 34.57%, 30.37%, and 39.00%, respectively. EGCG decreased ALT, AST, and ALP by 49.59%, 45.52%, and 41.10%, respectively, compared with HFD. Relative to Lipanthyl, EGCG showed lower ALT and AST values by 22.96% and 21.75%, respectively, while ALP was numerically lower by 3.44% (*p* < 0.05).

Sodium selenite decreased ALT, AST, and ALP by 38.20%, 40.02%, and 36.32%, respectively, compared with HFD. Relative to Lipanthyl, sodium selenite showed a lower AST value by 13.86%, whereas ALT and ALP were close to or slightly higher than the Lipanthyl group. EGCG-SeNPs decreased ALT, AST, and ALP by 56.01%, 57.68%, and 47.82%, respectively, compared with HFD. Compared with Lipanthyl, EGCG-SeNPs showed lower ALT, AST, and ALP values by 32.77%, 39.22%, and 14.45%, respectively.

### 2.4. Cardiac Enzymes: CK-MB and Troponin (cTnI)

**Figure 5:**
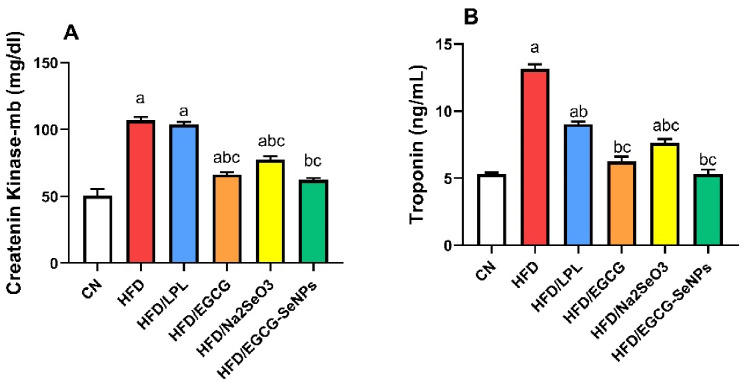
Effect of Lipanthyl, EGCG, sodium selenite, and EGCG-SeNPs on serum cardiac injury biomarkers in HFD-fed rats. (**A**) CK-MB and (**B**) cTnI levels in different experimental groups. Data are presented as mean ± SEM. Statistical analysis was performed using one-way ANOVA followed by Tukey’s post hoc test. a = vs. Control; b = vs. HFD; c = vs. HFD/Lipanthyl (*p* < 0.05).

As illustrated in Figure 5, HFD feeding increased serum CK-MB from 50.61 ± 4.99 to 106.90 ± 2.51 and cTnI from 5.30 ± 0.12 to 13.14 ± 0.35, representing increases of 111.24% and 147.81%, respectively, compared with the control group.

Compared with HFD, Lipanthyl produced a small numerical reduction in CK-MB of 2.79% and decreased cTnI by 31.19%. EGCG decreased CK-MB and cTnI by 37.83% and 52.40%, respectively, compared with HFD. Relative to Lipanthyl, EGCG showed lower CK-MB and cTnI values by 36.04% and 30.83%, respectively (*p* < 0.05).

Sodium selenite decreased CK-MB and cTnI by 27.61% and 42.07%, respectively, compared with HFD. Relative to Lipanthyl, sodium selenite showed lower CK-MB and cTnI values by 25.54% and 15.81%, respectively. EGCG-SeNPs decreased CK-MB and cTnI by 41.63% and 59.57%, respectively, compared with HFD. Compared with Lipanthyl, EGCG-SeNPs showed lower CK-MB and cTnI values by 39.96% and 41.24%, respectively.

### 2.5. Investigation of Oxidative and Antioxidant Biomarkers

**Figure 6:**
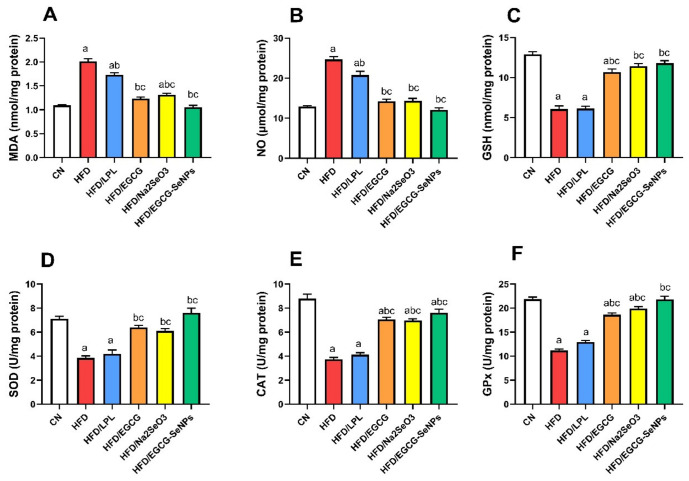
Effect of selenium, EGCG, and EGCG-SeNPs on oxidative stress biomarkers in liver tissue of HFD-fed rats. (**A**) MDA, (**B**) NO, (**C**) GSH, (**D**) SOD, (**E**) CAT, and (**F**) GPx. Data are presented as mean ± SEM (n = 7). One-way ANOVA followed by Tukey’s post hoc test. a = vs. Control; b = vs. HFD; c = vs. HFD/LPL (*p* < 0.05).

As shown in Figure 6, HFD feeding disrupted hepatic redox-related biomarkers. Compared with the control group, MDA increased from 1.09 ± 0.02 to 2.01 ± 0.06, and NO increased from 12.94 ± 0.21 to 24.70 ± 0.65, corresponding to increases of 84.25% and 90.92%, respectively. In contrast, GSH decreased from 12.89 ± 0.34 to 6.07 ± 0.40, SOD decreased from 7.11 ± 0.20 to 3.86 ± 0.17, CAT decreased from 8.79 ± 0.36 to 3.73 ± 0.16, and GPx decreased from 21.86 ± 0.45 to 11.20 ± 0.32, representing reductions of 52.92%, 45.74%, 57.51%, and 48.76%, respectively (*p* < 0.05).

Compared with HFD, Lipanthyl decreased MDA and NO by 13.82% and 15.82%, respectively, while producing limited numerical increases in GSH, SOD, CAT, and GPx of 0.92%, 9.02%, 10.61%, and 15.71%, respectively. EGCG decreased MDA and NO by 38.67% and 42.23%, respectively, and increased GSH, SOD, CAT, and GPx by 75.75%, 65.73%, 88.81%, and 66.29%, respectively, compared with HFD.

Sodium selenite decreased MDA and NO by 34.69% and 41.93%, respectively, and increased GSH, SOD, CAT, and GPx by 88.43%, 58.32%, 86.40%, and 77.50%, respectively, compared with HFD. EGCG-SeNPs decreased MDA and NO by 47.42% and 50.97%, respectively, and increased GSH, SOD, CAT, and GPx by 94.60%, 97.36%, 104.18%, and 95.00%, respectively, compared with HFD. Relative to Lipanthyl, EGCG-SeNPs showed lower MDA and NO values by 38.99% and 41.75%, respectively, and higher GSH, SOD, CAT, and GPx values by 92.82%, 81.03%, 84.60%, and 68.52%, respectively.

### 2.6. Evaluation of Hepatic Keap1/Nrf2 Antioxidant Signaling

**Figure 7:**
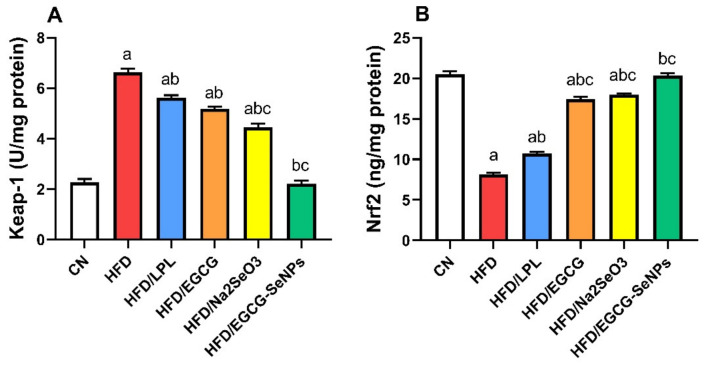
Effects of EGCG-SeNPs on hepatic Keap1/Nrf2 signaling in HFD-fed rats. Hepatic Keap1 (**A**) and Nrf2 (**B**). Data are presented as mean ± SEM (n = 7). Different superscript letters indicate significant differences at *p* < 0.05: a, versus control; b, versus HFD; and c, versus HFD/Lipanthyl (*p* < 0.05).

As shown in Figure 7, HFD feeding increased Keap1 from 2.26 ± 0.14 to 6.63 ± 0.16, representing a 193.19% increase compared with the control group. In contrast, Nrf2 decreased from 20.52 ± 0.37 to 8.16 ± 0.17, corresponding to a 60.23% reduction (*p* < 0.05).

Compared with HFD, Lipanthyl decreased Keap1 by 15.18% and increased Nrf2 by 31.37%. EGCG decreased Keap1 by 21.73% and increased Nrf2 by 113.73% compared with HFD. Sodium selenite decreased Keap1 by 32.87% and increased Nrf2 by 120.10% compared with HFD. EGCG-SeNPs decreased Keap1 by 66.65% and increased Nrf2 by 149.26% compared with HFD. Relative to Lipanthyl, EGCG-SeNPs showed a 60.68% lower Keap1 value and an 89.74% higher Nrf2 value.

### 2.7. Investigation of Inflammatory Biomarkers

**Figure 8:**
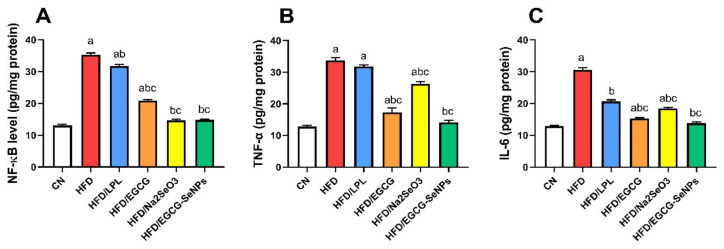
Effect of selenium, EGCG, and EGCG-SeNPs on inflammatory mediators in high-fat diet-induced hepatic stress. (**A**) NF-kB, (**B**) TNF-α, and (**C**) IL-6 concentrations in liver tissue. Data are presented as mean ± SEM (n = 7). One-way ANOVA followed by Tukey’s post hoc test. a = vs. Control; b = vs. HFD; c = vs. HFD/LPL (*p* < 0.05).

As illustrated in Figure 8, HFD feeding increased hepatic inflammatory markers compared with the control group. NF-κB increased from 13.04 ± 0.43 to 35.36 ± 0.51, TNF-α increased from 12.82 ± 0.37 to 33.72 ± 0.89, and IL-6 increased from 12.89 ± 0.19 to 30.56 ± 0.69, corresponding to increases of 171.17%, 163.01%, and 137.02%, respectively.

Compared with HFD, Lipanthyl decreased NF-κB, TNF-α, and IL-6 by 10.18%, 5.65%, and 32.30%, respectively. EGCG decreased NF-κB, TNF-α, and IL-6 by 41.06%, 48.67%, and 49.76%, respectively, compared with HFD. Relative to Lipanthyl, EGCG showed lower NF-κB, TNF-α, and IL-6 values by 34.38%, 45.60%, and 25.80%, respectively (*p* < 0.05).

Sodium selenite decreased NF-κB, TNF-α, and IL-6 by 58.48%, 21.84%, and 39.53%, respectively, compared with HFD. EGCG-SeNPs decreased NF-κB, TNF-α, and IL-6 by 58.14%, 58.03%, and 54.88%, respectively, compared with HFD. Relative to Lipanthyl, EGCG-SeNPs showed reductions in NF-κB, TNF-α, and IL-6 by 53.40%, 55.52%, and 33.36%, respectively.

### 2.8. Modulation of Hepatic Lipogenic and Fatty Acid Oxidation

**Figure 9:**
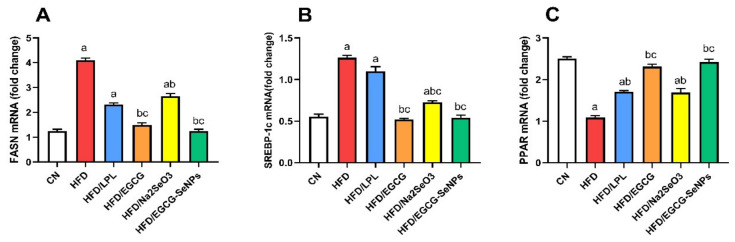
Effect of selenium, EGCG, and EGCG-SeNPs on hepatic lipogenic and oxidative gene expression. (**A**) FASN, (**B**) PPAR-α, and (**C**) SREBP-1 mRNA fold change in liver tissue. Data are presented as mean ± SEM (n = 7). One-way ANOVA followed by Tukey’s post hoc test. a = vs. Control; b = vs. HFD; c = vs. HFD/LPL (*p* < 0.05).

As shown in Figure 9, HFD feeding altered the hepatic expression of lipid metabolism-related genes. FASN expression increased from 1.24 ± 0.08 to 4.10 ± 0.09, and SREBP-1c expression increased from 0.55 ± 0.03 to 1.26 ± 0.03, representing increases of 229.95% and 127.80%, respectively. In contrast, PPAR-α expression decreased from 2.50 ± 0.05 to 1.09 ± 0.05, corresponding to a 56.56% reduction compared with the control group (*p <* 0.05).

Compared with HFD, Lipanthyl decreased FASN and SREBP-1c by 43.39% and 12.84%, respectively, and increased PPAR-α by 57.27%. EGCG decreased FASN and SREBP-1c by 63.74% and 58.99%, respectively, and increased PPAR-α by 112.80% compared with HFD. Relative to Lipanthyl, EGCG showed lower FASN and SREBP-1c values by 35.95% and 52.95%, respectively, and a higher PPAR-α value by 35.30%.

Sodium selenite decreased FASN and SREBP-1c by 35.40% and 42.42%, respectively, and increased PPAR-α by 55.43% compared with HFD. EGCG-SeNPs decreased FASN and SREBP-1c by 69.64% and 57.05%, respectively, and increased PPAR-α by 123.02% compared with HFD. Relative to Lipanthyl, EGCG-SeNPs showed lower FASN and SREBP-1c values by 46.38% and 50.73%, respectively, and a higher PPAR-α value by 41.80%.

### 2.9. Evaluation of Apoptotic Activity

**Figure 10:**
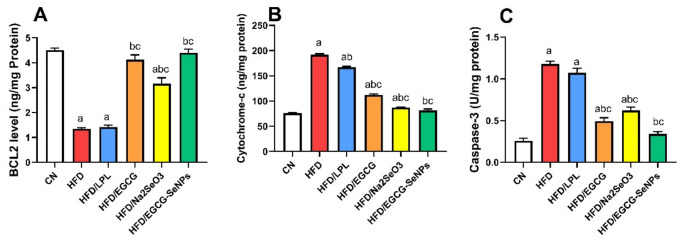
Effect of selenium, EGCG, and EGCG-SeNPs on hepatic apoptotic markers in HFD-fed rats. (**A**) Bcl-2 expression, (**B**) cytochrome c, and (**C**) caspase-3 activity. Data are presented as mean ± SEM (n = 7). One-way ANOVA followed by Tukey’s post hoc test. a = vs. Control; b = vs. HFD; c = vs. HFD/LPL (*p* < 0.05).

As illustrated in Figure 10, HFD feeding shifted hepatic apoptotic markers compared with the control group. Bcl-2 decreased from 4.51 ± 0.08 to 1.35 ± 0.04, representing a 70.11% reduction. Cytochrome c increased from 75.76 ± 1.28 to 191.80 ± 2.15, and caspase-3 increased from 0.26 ± 0.03 to 1.18 ± 0.03, corresponding to increases of 153.17% and 359.40%, respectively.

Compared with HFD, Lipanthyl produced a small numerical increase in Bcl-2 of 5.34%, while cytochrome c and caspase-3 decreased by 12.84% and 9.00%, respectively. EGCG increased Bcl-2 by 205.93% and decreased cytochrome c and caspase-3 by 41.45% and 58.23%, respectively, compared with HFD. Relative to Lipanthyl, EGCG showed a higher Bcl-2 value by 190.42% and lower cytochrome c and caspase-3 values by 32.83% and 54.10%, respectively (*p* < 0.05).

Sodium selenite increased Bcl-2 by 134.12% and decreased cytochrome c and caspase-3 by 54.70% and 47.20%, respectively, compared with HFD. EGCG-SeNPs increased Bcl-2 by 226.56% and decreased cytochrome c and caspase-3 by 57.56% and 71.14%, respectively, compared with HFD. Relative to Lipanthyl, EGCG-SeNPs showed a higher Bcl-2 value by 210.00% and lower cytochrome c and caspase-3 values by 51.31% and 68.28%, respectively.

### 2.10. Evaluation of Mitochondrial Homeostasis-Related Gene Expression

**Figure 11:**
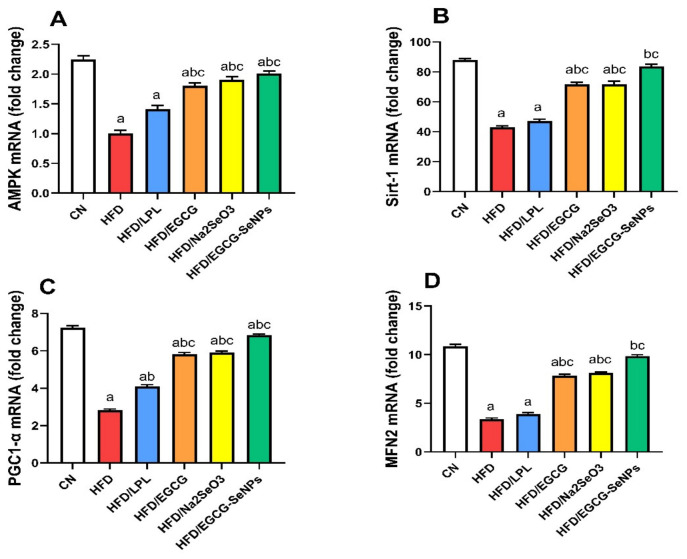
Effects of EGCG-functionalized selenium nanoparticles (EGCG-SeNPs) on hepatic metabolic and mitochondrial homeostasis-related gene expression in HFD-fed rats. Relative mRNA expression of AMPK (**A**), Sirt1 (**B**), PGC-1α (**C**), and MFN2 (**D**). Data are presented as mean ± SEM (n = 7). Statistical significance was determined using one-way ANOVA followed by Tukey’s post hoc test. Superscript letters denote significant differences at *p* < 0.05: a, versus CN; b, versus HFD; and c, versus HFD/LPL (*p* < 0.05).

As shown in Figure 11, HFD feeding decreased the hepatic expression of mitochondrial homeostasis-related genes. AMPK expression decreased from 2.25 ± 0.06 to 1.00 ± 0.06, PGC-1α decreased from 7.24 ± 0.10 to 2.82 ± 0.08, and MFN2 decreased from 10.86 ± 0.20 to 3.37 ± 0.12, corresponding to reductions of 55.48%, 61.09%, and 68.99%, respectively, compared with the control group.

Compared with HFD, Lipanthyl increased AMPK, PGC-1α, and MFN2 by 41.40%, 45.67%, and 16.39%, respectively. EGCG increased AMPK, PGC-1α, and MFN2 by 80.60%, 106.96%, and 132.36%, respectively, compared with HFD. Relative to Lipanthyl, EGCG showed higher AMPK, PGC-1α, and MFN2 values by 27.72%, 42.08%, and 99.64%, respectively (*p* < 0.05).

Sodium selenite increased AMPK, PGC-1α, and MFN2 by 90.80%, 109.94%, and 141.15%, respectively, compared with HFD. EGCG-SeNPs increased AMPK, PGC-1α, and MFN2 by 101.40%, 142.68%, and 192.28%, respectively, compared with HFD. Relative to Lipanthyl, EGCG-SeNPs showed higher AMPK, PGC-1α, and MFN2 values by 42.43%, 66.60%, and 151.12%, respectively.

### 2.11. Histopathological Analysis

**Figure 12:**
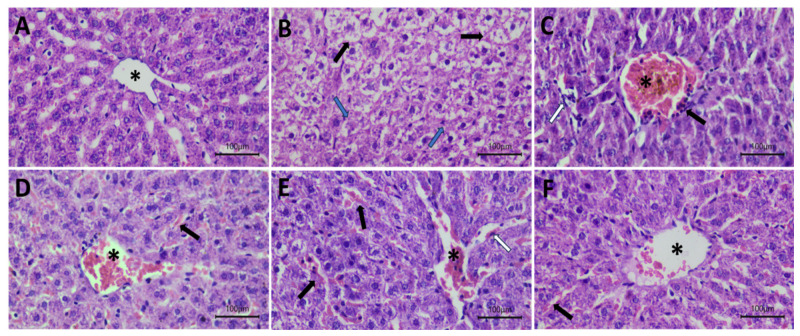
Photomicrograph of H&E-stained hepatic tissues of all study groups. (**A**) The control group showed a central vein (*) and radially arranged hepatocytes (black arrow) with interlobular sinusoids (blue arrow). (**B**) The HFD group showed ballooned, degenerated hepatocytes (black arrow) and apoptotic hepatocytes with pyknosis and karyolysis (blue arrow). (**C**) The HFD/LPL group showed interlobular inflammatory cellular infiltration (white arrow), perivascular inflammatory cellular infiltration (black arrow), and congested central vein (*). (**D**) The HFD/EGCG group showed a congested central vein (*) and sinusoidal hemorrhage (black arrow). (**E**) The HFD/Na_2_SeO_3_ group showed sinusoidal edema (white arrow), congested central vein (*), and sinusoidal congestion (black arrow). (**F**) The HFD/EGCG-SeNP group revealed improved histological structure with a normal central vein (*) and mildly congested sinusoids (black arrow). H&E, ×400.

Histopathological examination revealed progressive amelioration of hepatic damage across treatment groups. The control group exhibited normal hepatic architecture characterized by intact hepatic lobules with organized hepatocyte cords, centrally located nuclei with prominent nucleoli, and appropriately distributed Kupffer cells within hepatic sinusoids, consistent with healthy liver parenchyma. High-fat diet (HFD) administration induced severe hepatic pathology, manifested by widespread hydropic degeneration of hepatocytes accompanied by scattered apoptotic bodies, indicative of substantial oxidative stress and cellular injury. The HFD/LPL co-treatment group showed exacerbated pathology with marked central vein congestion, extensive extravascular inflammatory cell infiltration, interlobular hemorrhage, and diffuse interlobular inflammatory cell accumulation, reflecting severe inflammatory responses and microvascular dysfunction. In contrast, pharmacological interventions significantly attenuated hepatic damage. The HFD/EGCG group demonstrated moderate improvement, characterized by reduced hemorrhagic foci and controlled central vein congestion. Similarly, HFD/Na_2_SeO_3_ treatment produced comparable protective effects, though with persistent irregular central vein architecture and interlobular hemorrhage. Notably, the HFD/EGCG-SeNP combination group exhibited the most pronounced hepatoprotection, displaying near-complete restoration of liver architecture with only mild interlobular hemorrhage and minimal inflammatory infiltration (Figure 12).

These findings suggest synergistic hepatoprotective mechanisms of epigallocatechin-3-gallate and selenium nanoparticles against HFD-induced hepatic injury, with the combined therapy demonstrating superior efficacy in preserving hepatic structural integrity and preventing inflammatory-hemorrhagic complications.

### 2.12. Immunohistochemical Analysis

**Figure 13:**
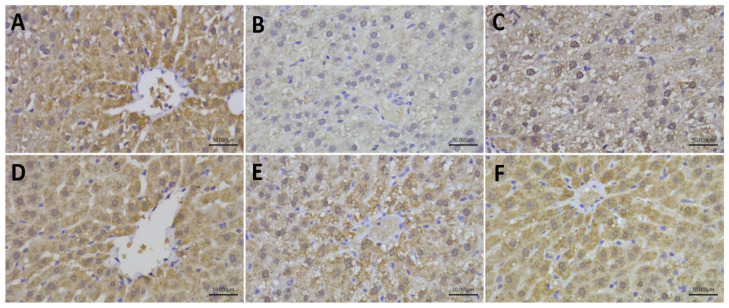
Impact of EGCG-SeNPs on hepatic immunoreactivity of Nrf2 in HFD-induced hepatic injury. The reactivity of Nrf2 was apparent in the cells as a brown color (arrow) (DAB, ×400). (**A**) Control, (**D**) HFD/EGCG, and (**F**) HFD/EGCG-SeNP groups demonstrated strong Nrf2 staining. (**C**) HFD/LPL and (**E**) HFD/Na_2_SeO_3_ groups revealed moderate Nrf2 immunostaining. In contrast, (**B**) HFD revealed weak Nrf2 staining.

Immunohistochemical analysis of hepatic Nrf2 expression revealed a striking correlation between antioxidant transcription factor activation and hepatoprotection across treatment groups. The control group demonstrated marked Nrf2 immunoreactivity, consistent with baseline antioxidant defense mechanisms in healthy liver tissue. High-fat diet exposure significantly suppressed Nrf2 expression, with the HFD group showing weak immunoreaction, indicating impaired activation of the Nrf2-dependent antioxidant response element pathway and compromised cellular defense against oxidative stress. This diminished Nrf2 signaling correlated with the severe hepatocellular damage, hydropic degeneration, and apoptosis observed in HFD-treated animals. The HFD/LPL and HFD/Na_2_SeO_3_ groups exhibited moderate Nrf2 immunostaining, suggesting partial restoration of the antioxidant defense pathway, though remaining below control levels. Notably, both HFD/EGCG and HFD/EGCG-SeNP treatment groups demonstrated marked Nrf2 expression comparable to controls, indicating robust reactivation of Nrf2-mediated antioxidant signaling. The marked Nrf2 expression in HFD/EGCG and HFD/EGCG-SeNP groups directly correlated with superior hepatoprotection and minimal inflammatory-hemorrhagic pathology, whereas moderate Nrf2 activation in HFD/LPL and HFD/Na_2_SeO_3_ groups corresponded to intermediate histological improvements (Figure 13).

These findings suggest that EGCG and selenium nanoparticles, particularly in combination, potently activate the Nrf2/ARE antioxidant defense pathway, thereby ameliorating HFD-induced hepatic oxidative stress and inflammation through enhanced cytoprotective gene expression.

## 3. Discussion

**Figure 14:**
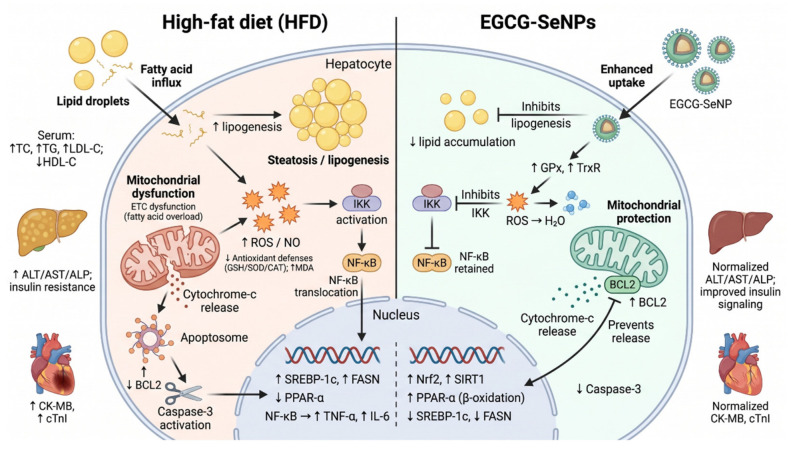
Intracellular mechanism of HFD-induced lipotoxicity and multi-targeted rescue by EGCG-SeNPs in hepatocytes. High-fat diet feeding induces a serial pathogenic cascade within hepatocytes: fatty acid overload and mitochondrial ETC dysfunction trigger excessive ROS/NO generation, which simultaneously depletes endogenous antioxidants (GSH, SOD, CAT) and activates the redox-sensitive NF-κB inflammatory axis. This oxidative and inflammatory state down-regulates *PPAR*α, stimulates *SREBP-1c*/*FASN* lipogenesis, and destabilizes mitochondrial membranes to release cytochrome-c, culminating in caspase-3-mediated apoptosis, hepatic steatosis, and secondary cardiac injury (CK-MB, cTnI). Conversely, EGCG-SeNPs utilize an advanced nano-delivery platform to simultaneously suppress lipogenesis, activate Nrf2/SIRT1-dependent antioxidant defenses, arrest NF-κB nuclear translocation, and up-regulate BCL2 expression, completely restoring tissue architecture and cardio-hepatic homeostasis. Created in BioRender. Ahms, A. BioRender.com/s96dv2v.

High-fat diet (HFD)-induced hyperlipidemia constitutes a multifactorial metabolic insult characterized by systemic dyslipidemia, hepatic steatosis, oxidative stress, chronic inflammation, and apoptotic activation [19]. In this study, HFD feeding significantly increased serum total cholesterol, triglycerides, and LDL-C, while decreasing HDL-C, consistent with an imbalance in hepatic lipid synthesis and fatty acid catabolism. At the transcriptional level, key lipogenic genes—FASN and SREBP-1c—were markedly upregulated, while PPAR-α, a master regulator of fatty acid oxidation, was suppressed. Mechanistically, this gene expression pattern reflects a metabolic shift favoring lipid accumulation, impaired β-oxidation, and decreased lipid clearance from the liver, creating a hepatocellular environment predisposed to oxidative and inflammatory damage (Figure 14).

The dyslipidemic state triggered profound oxidative stress. Elevated MDA and NO levels indicate enhanced lipid peroxidation and nitrosative stress, while concomitant reductions in endogenous antioxidants GSH, SOD, and CAT reflect depletion of the hepatocellular defense system [20]. Excessive ROS generation is mechanistically linked to fatty acid overload and mitochondrial electron transport chain dysfunction, which together propagate peroxidative damage to lipids, proteins, and DNA. This oxidative imbalance not only damages cellular components but also amplifies the activation of redox-sensitive transcription factors such as NF-κB, thereby promoting pro-inflammatory gene expression and establishing a feed-forward cycle between oxidative stress and inflammation (Figure 14) [21,22,23].

The redox imbalance induced by HFD was further substantiated by disruption of the Keap1/Nrf2 antioxidant regulatory axis. Increased hepatic Keap1 levels, accompanied by reduced Nrf2, indicate impaired mobilization of endogenous cytoprotective responses under sustained lipid-derived oxidative stress [24]. This alteration is mechanistically consistent with the observed accumulation of MDA and NO and the depletion of GSH, SOD, CAT, and GPx activity [24,25]. Notably, EGCG-SeNPs produced the most effective correction of this antioxidant imbalance by reducing Keap1, restoring Nrf2 levels, and improving the enzymatic and non-enzymatic antioxidant profile. The recovery of GPx is particularly relevant to the selenium-based nature of the formulation, as selenium is required for the functional activity of glutathione peroxidase enzymes involved in peroxide detoxification and protection against lipid oxidative damage [10,26]. Thus, EGCG-SeNPs appear to combine the radical-scavenging properties of EGCG with selenium-supported antioxidant defense, thereby providing a plausible explanation for their superior ability to restore hepatic redox homeostasis.

The observed correlation between MDA/NO accumulation and dysregulated lipid profiles supports the hypothesis that oxidative stress is both a consequence of lipid overload and a driver of further hepatic lipid dysregulation, as ROS can induce SREBP-1c activation and impair PPAR-α function, thereby compounding lipotoxicity. This mechanistic insight explains the synergistic interdependence between dyslipidemia and oxidative injury [7,27].

HFD-induced oxidative stress directly engages inflammatory pathways. NF-κB activation leads to increased transcription of TNF-α, IL-6, and other pro-inflammatory mediators, establishing chronic low-grade inflammation within the liver [28]. Mechanistically, ROS modulate IκB kinase complex activity [29], facilitating NF-κB nuclear translocation and transcriptional activation of inflammatory cytokines [30]. These cytokines, in turn, exacerbate hepatic injury, impair insulin signaling, and further promote lipid accumulation via upregulation of lipogenic transcription factors. The sustained activation of NF-κB not only contributes to local tissue injury but also promotes systemic inflammatory responses, as evidenced by elevated cardiac injury markers CK-MB and cTnI, highlighting cross-organ effects of HFD-induced oxidative-inflammation crosstalk [31].

The progression of oxidative and inflammatory injury was accompanied by a clear disturbance in hepatic apoptotic homeostasis. HFD reduced Bcl-2 levels while increasing cytochrome c and caspase-3, indicating enhanced mitochondria-associated apoptotic signaling [32]. Bcl-2 is a key regulator of mitochondrial membrane stability, whereas increased tissue cytochrome c is consistent with greater engagement of the mitochondrial apoptotic pathway and downstream caspase activation. The present results therefore connect HFD-induced oxidative stress and impaired mitochondrial adaptation with hepatocellular apoptotic injury. EGCG-SeNPs markedly restored Bcl-2 levels and suppressed the elevations in cytochrome c and caspase-3 more effectively than either epigallocatechin-3-gallate or sodium selenite alone. This protective profile suggests that restoration of antioxidant capacity and metabolic–mitochondrial regulation by EGCG-SeNPs ultimately limits the progression of apoptotic hepatic damage, in agreement with the improved histological appearance of liver tissue in the treated group [33,34].

**Figure 15:**
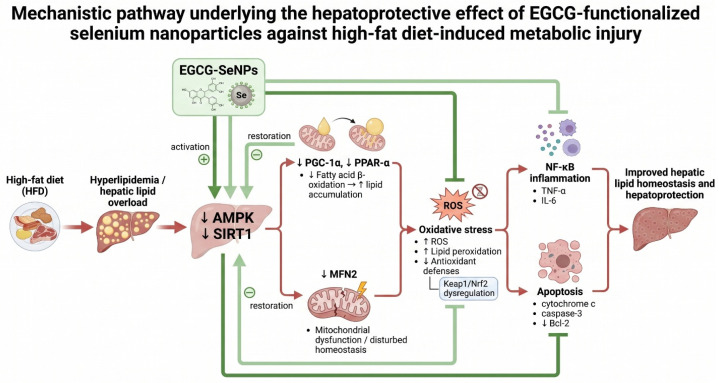
Proposed mechanism of the hepatoprotective effect of EGCG-functionalized selenium nanoparticles (EGCG-SeNPs) against HFD-induced metabolic injury.

The transcriptional alterations observed in the present study further indicate that HFD-associated lipid dysregulation was accompanied by impairment of hepatic metabolic and mitochondrial adaptive responses. HFD markedly suppressed the mRNA expression of *Ampk*, *Sirt1*, *Ppargc1a* (PGC-1α), and *Mfn2*, suggesting reduced energy-sensing capacity, weakened mitochondrial oxidative programming, and disrupted mitochondrial dynamics. AMPK and SIRT1 are functionally interconnected regulators of hepatic lipid utilization [24,35], while PGC-1α acts as a downstream metabolic coactivator that supports mitochondrial oxidative capacity and cooperates with PPAR-α to promote fatty-acid catabolism [36,37]. In parallel, MFN2 contributes to mitochondrial fusion and functional network integrity; therefore, its suppression may favor mitochondrial fragmentation and lipotoxic stress [38]. The marked restoration of these transcripts by EGCG-SeNPs, together with recovery of *Ppara* and suppression of *Srebf1* and *Fasn*, supports a coordinated mechanism in which the nanoformulation improves lipid homeostasis while preserving mitochondrial adaptation. Nevertheless, because these endpoints were evaluated at the mRNA level, the present data support transcriptional regulation of the AMPK/SIRT1/PGC-1α/MFN2-associated axis rather than definitive confirmation of pathway activation at the protein or phosphorylation level (Figure 15).

Fenofibrate partially mitigated lipid dysregulation and oxidative stress, primarily by activating PPAR-α and enhancing fatty acid oxidation [36]. However, residual oxidative stress and incomplete normalization of lipid and apoptotic markers highlight the limitations of single-target pharmacological interventions in addressing complex HFD-induced pathology.

Epigallocatechin-3-gallate (EGCG) supplementation provided enhanced protection via its polyphenolic structure, which enables direct ROS scavenging, inhibition of SREBP-1c-mediated lipogenesis, and partial suppression of NF-κB-driven inflammation [39]. This multifaceted action improved hepatic lipid profiles, restored antioxidant enzyme activity, and reduced apoptotic signaling [40,41].

Selenium supplementation conferred additional benefits by supporting the activity of selenoproteins such as glutathione peroxidase (GPx) and thioredoxin reductase (TrxR), which detoxify ROS and maintain redox homeostasis. Selenium also modulated transcriptional regulators of lipid metabolism, partially restoring PPAR-α activity and reducing lipotoxic stress [42].

EGCG-functionalized selenium nanoparticles (EGCG-SeNPs) demonstrated the most profound therapeutic effects. Nanoparticle-mediated co-delivery enhances bioavailability and cellular uptake, producing synergistic modulation of oxidative, inflammatory, and lipogenic pathways. EGCG-SeNPs downregulated FASN and SREBP-1c, upregulated PPAR-α, and activated Nrf2/Keap1 and SIRT1 signaling, restoring endogenous antioxidant capacity and improving redox balance. Simultaneously, NF-κB signaling was attenuated, reducing TNF-α and IL-6 levels. The combined antioxidant and anti-inflammatory actions stabilized mitochondrial membranes, increased BCL2 expression, and decreased caspase-3 activation, preserving hepatocyte integrity and functional architecture (Figure 15) [10,13,43].

Beyond hepatic protection, EGCG-SeNPs exerted cardioprotective effects, normalizing CK-MB and cTnI levels. These outcomes suggest that attenuation of systemic oxidative and inflammatory stress reduces cardiac injury secondary to hyperlipidemia. The findings highlight the multi-organ benefits of a nanoparticle-based strategy that simultaneously targets dyslipidemia, oxidative stress, inflammation, and apoptosis [13].

Collectively, the data reveal a serial mechanistic cascade underlying HFD-induced metabolic derangements: lipid accumulation → ROS overproduction → NF-κB-mediated inflammation → apoptotic activation → tissue injury. EGCG-SeNPs intervene at multiple nodes, restoring lipid homeostasis, enhancing Nrf2/SIRT1-dependent antioxidant defenses, suppressing NF-κB-driven inflammation, and stabilizing mitochondrial apoptotic pathways. This multi-targeted approach surpasses the effects of EGCG or selenium alone, providing a potent and translationally relevant therapeutic strategy for HFD-induced metabolic and organ dysfunction (Figure 15).

This study has several limitations. The 6-week HFD induction period and 4-week treatment window represent a relatively brief timeframe that may not capture chronic metabolic adaptations, disease progression, or late-stage therapeutic responses. Whole-body lipid metabolism studies were not conducted, limiting mechanistic insights into lipogenesis versus clearance pathways. The study did not determine plasma concentrations, tissue distribution, or clearance rates of free EGCG or selenium following administration of EGCG-SeNPs.

A major limitation of the present study is the absence of separate control groups receiving blank nanoparticles or non-EGCG-functionalized selenium nanoparticles. Therefore, although EGCG-SeNPs produced greater improvement than free EGCG and sodium selenite under the current experimental conditions, the specific contribution of the nanoparticle carrier and the independent effect of EGCG functionalization cannot be fully distinguished. The present study was primarily designed to evaluate the protective efficacy of EGCG-SeNPs against HFD-induced hepatic injury, rather than to provide a complete formulation-specific safety or carrier-control assessment. Previous studies have reported the relative biocompatibility, stability, and biological activity of EGCG-, selenium-, and EGCG/SeNP-based nanoformulations.

Future studies should include targeted or untargeted lipidomics to assess major lipid classes, including fatty acids, sphingolipids, phospholipids, glycerolipids, sterols, and lipid mediators, in order to provide a deeper mechanistic understanding of how EGCG-SeNPs regulate lipid metabolism under HFD-induced hepatic lipotoxicity.

## 4. Materials and Methods

### 4.1. Preparation and Characterization of EGCG-Functionalized Selenium Nanoparticles

**Figure 16:**
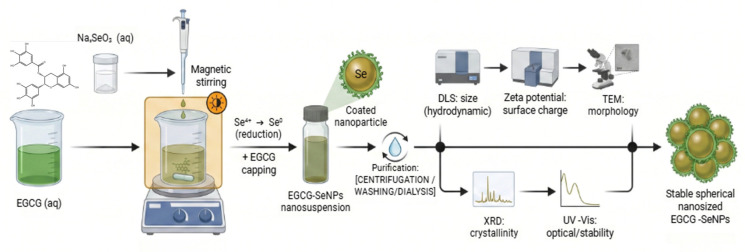
Green synthesis of EGCG-functionalized selenium nanoparticles (EGCG-SeNPs). Created in BioRender. Ahms, A. BioRender.com/s96dv2v.

Epigallocatechin-3-gallate-functionalized selenium nanoparticles (EGCG-SeNPs) were synthesized following the procedure reported by Alam-ElDein et al. (2026) [13], with modifications appropriate to the current experimental design. An aqueous solution of sodium selenite (Na_2_SeO_3_; 10 mM; 10 mL) was combined with an equal volume of EGCG solution (3.5 mg/mL; 10 mL). The resulting mixture was maintained under continuous magnetic stirring at room temperature for 12 h, allowing EGCG-mediated reduction of selenium ions and concurrent stabilization of the formed nanoparticles through surface capping, as shown in Figure 16. The generated EGCG-SeNPs were then used for physicochemical characterization and subsequently in vivo administration.

#### 4.1.1. Zeta Potential and Particle Size

The surface charge and hydrodynamic properties of EGCG-synthesized selenium nanoparticles (EGCG-SeNPs) were determined by zeta potential and dynamic light scattering (DLS), respectively, using a Zetasizer Nano ZS90 (Malvern Panalytical, Malvern, UK) at the Faculty of Engineering, Capital University.

#### 4.1.2. Transmission Electron Microscopy (TEM)

Morphological characteristics and structural integrity were further examined using high-resolution TEM (JEOL, Tokyo, Japan) at Al-Azhar University, Cairo, Egypt. Nanoparticle suspensions were deposited onto carbon-coated copper grids, and TEM imaging confirmed uniform nanoscale dimensions and consistent structural morphology of the synthesized SeNPs.

#### 4.1.3. X-Ray Diffraction Analysis of EGCG-SeNPs (XRD)

The structural characteristics of epigallocatechin gallate-mediated selenium nanoparticles (EGCG-SeNPs) were evaluated using X-ray diffraction (XRD) analysis at Al-Azhar University, Cairo, Egypt. The dried nanoparticle formulation was subjected to XRD scanning, and the generated diffraction profile was analyzed to identify the position, intensity, full width at half maximum (FWHM), and corresponding interplanar spacing (d-spacing) of the detected peaks. The obtained diffraction data were used to assess the structural organization and crystallinity pattern of the synthesized EGCG-SeNPs.

#### 4.1.4. UV–Visible Stability Assessment of EGCG-SeNPs

The temporal stability of epigallocatechin gallate-mediated selenium nanoparticles (EGCG-SeNPs) was evaluated by ultraviolet–visible (UV–Vis) spectrophotometry at Badr University in Cairo (BUC), Cairo, Egypt. Freshly prepared EGCG-SeNPs were initially scanned over the wavelength range of 200–800 nm, and the analysis was repeated at monthly intervals during storage to monitor changes in their optical profile. Stability was assessed by comparing the position and intensity pattern of the characteristic absorption bands of fresh EGCG-SeNPs with those recorded after storage.

The synthesis conditions were selected after preliminary optimization trials aimed at obtaining a stable and uniformly dispersed EGCG-SeNP colloidal formulation. Successful nanoparticle formation was monitored by the characteristic color change of the reaction mixture and confirmed by UV–Vis spectrophotometry, DLS particle-size analysis, zeta potential measurement, and TEM examination. The final preparation conditions were chosen based on the formation of nanosized particles with good dispersion, high surface charge, minimal aggregation, and acceptable storage stability. Because the formulation was used as a colloidal nanosuspension for characterization and animal administration, the gravimetric dried-product yield was not calculated in the present study.

### 4.2. Experimental Animals and Experimental Design

This study was designed to evaluate the potential therapeutic efficacy of green-synthesized nanoparticles prepared using Epigallocatechin Gallate (EGCG-SeNPs) in ameliorating diet-induced hyperlipidemia. A total of forty-two adult male albino rats (250–280 g) were utilized, randomly allocated into six experimental groups, with seven animals per group. The rats were housed under controlled environmental conditions (22 ± 2 °C, 50–60% relative humidity, and a 12:12 h light/dark cycle) and provided free access to water and food. The animal study protocol was approved by the Ethics Committee of the School of Biotechnology, Badr University, in Cairo, Egypt (protocol code BUC-IACUC/BIOT/136/A/2026 and 14 February 2026 as the date of approval).

All treatments, including Lipanthyl, EGCG, sodium selenite, and EGCG-SeNPs, were freshly prepared and administered once daily by oral gavage at the assigned doses. The control and untreated HFD groups received the corresponding vehicle by oral gavage.

The grouping of animals was structured as follows:

(1) Control Group (CN, n = 7): Animals of this group were exposed to standard conditions for consecutive days. At day 15, animals of this group received normal saline solution (0.9% NaCl) for 30 consecutive days.
(2) High-Fat Diet Group (HFD, n = 7): Animals of this group were fed a high-fat diet (HFD) to induce hyperlipidemia for 6 weeks. At day 15, animals of this group received normal saline solution (0.9% NaCl) orally for 30 consecutive days.
(3) Standard Drug Group (HFD/LPL, n = 7): Animals of this group were fed a high-fat diet (HFD) to induce hyperlipidemia for 6 weeks. At day 15, animals of this group received daily oral administration of standard drug “LPL” at a dose of 200 mg/kg/day for 30 consecutive days [18,44].
(4) EGCG Group (HFD/EGCG, n = 7): Animals of this group were fed a high-fat diet (HFD) to induce hyperlipidemia for 6 weeks. At day 15, animals of this group received daily oral administration of Epigallocatechin Gallate “EGCG” at a dose of 200 mg/kg/day for 30 consecutive days [13].
(5) Sodium Selenite Group (HFD/Na_2_SeO_3_, n = 7): Animals of this group were fed a high-fat diet (HFD) to induce hyperlipidemia for 6 weeks. At day 15, animals of this group received daily oral administration of sodium selenite “Na_2_SeO_3_” at a dose of 0.5 mg/kg/day for 30 consecutive days [13].
(6) Selenium Nanoparticles Biosynthesized using Epigallocatechin Gallate Group (HFD/EGCG-SeNPs, n = 7): Animals of this group were fed a high-fat diet (HFD) to induce hyperlipidemia for 6 weeks. At day 15, animals of this group received daily oral administration of Se nanoparticles biosynthesized using EGCG “EGCG-SeNPs” at a dose of 0.5 mg/kg/day for 30 consecutive days [45,46].

The high-fat diet (HFD) was prepared according to a previously described atherogenic diet-induced hyperlipidemia model in Wistar rats. Briefly, the diet consisted of 75% commercially available Teklad TD.02028 Western purified atherogenic diet supplemented with 15% lard and 10% coconut oil. Compared with the standard laboratory chow, this HFD had a higher fat and caloric density and included atherogenic components such as cholesterol and cholic acid, thereby promoting dyslipidemia, hepatic lipid deposition, and liver injury. The control group received standard laboratory chow, whereas all HFD groups received the HFD for 6 weeks to induce experimental hyperlipidemia [47].

### 4.3. Power Analysis and Sample Size Justification

The sample size of n = 7 animals per group (total N = 42) was justified based on a priori power analysis, which indicated that this sample size provided >95% statistical power to detect the anticipated effect sizes (Cohen’s d ≥ 2.0) for the primary outcome measure (serum total cholesterol normalization). The very large effect sizes subsequently observed (d = 3.8–5.9 across biomarkers) indicate that the study achieved >99% statistical power for all major comparisons. The decision to employ n = 7 per group balanced the statistical objective of detecting treatment effects against the ethical imperative to minimize the total number of animals used (3R principles). The biological homogeneity of inbred laboratory rats maintained under controlled conditions, combined with the assessment of multiple independent biomarkers exhibiting consistent effects, collectively supported the adequacy of the sample size for the study objectives.

### 4.4. Tissue Collection and Sample Preparation

Animals were euthanized under deep anesthesia induced by intraperitoneal ketamine/xylazine (100 and 10 mg/kg body weight, respectively). The liver was promptly excised, carefully trimmed, and rinsed with ice-cold physiological saline to eliminate residual blood. The tissue was then divided into portions designated for biochemical, molecular, and immunohistochemical evaluations, as well as histological imaging.

For biochemical assessments, a portion of liver tissue was homogenized in 10 mM phosphate buffer (pH 7.4) to obtain a 10% (*w*/*v*) homogenate. The resulting mixture was centrifuged at 4500 rpm for 12 min, and the supernatant was collected for the determination of oxidative stress biomarkers, antioxidant enzyme activities, inflammatory mediators, apoptotic markers, and metabolic parameters, including glucose and insulin.

For histopathological and immunohistochemical analyses, liver samples were fixed in 10% neutral-buffered formalin, processed through routine paraffin embedding, and sectioned for subsequent staining and microscopic examination.

### 4.5. Serum Collection and Biochemical Analyses

Blood samples were allowed to clot at room temperature for 15 min and centrifuged at 3000 rpm at 4 °C to obtain serum for the assessment of hepatic enzymes, lipid profile, glucose, insulin, and insulin resistance indices. Liver homogenates (10% *w*/*v*) were prepared in phosphate-buffered saline (10 mM phosphate buffer, 138 mM NaCl, 2.7 mM KCl; pH 7.4), followed by centrifugation at 10,000 rpm for 10 min at 4 °C. The resulting supernatant was used for the determination of oxidative stress and inflammatory biomarkers. All parameters were quantified using commercially available ELISA and colorimetric assay kits according to the manufacturers’ protocols.

#### 4.5.1. Protein Determinations by ELISA

Protein biomarkers were quantified in serum or liver tissue homogenates using commercially available rat-specific ELISA kits according to the manufacturers’ instructions. Hepatic Keap1 and Nrf2 levels were determined using MyBioSource ELISA kits (San Diego, CA, USA) for Keap1 (Cat. No. MBS7218529) and Nrf2 (Cat. No. MBS752046), respectively. Hepatic GPx activity was measured using a MyBioSource ELISA kit (Cat. No. MBS744364). Inflammatory mediators were determined in liver tissue homogenates using ELISA kits for NF-κB (MyBioSource, Cat. No. MBS453975), TNF-α (MyBioSource, Cat. No. MBS700574), and IL-6 (MyBioSource, Cat. No. MBS2020158). Apoptotic markers were assessed using ELISA kits for cytochrome c (MyBioSource, Cat. No. MBS700786), Bcl-2 (FineTest, Cat. No. ER0762), and caspase-3 (MyBioSource, Cat. No. MBS261814).

#### 4.5.2. Assessment of Liver Enzyme Activities

Serum ALT, AST, and ALP activities were determined using commercially available colorimetric kits. ALT and AST levels were measured with commercial kits from BioVision (Cat. Nos. E4325-100 and E4321-100, respectively; Milpitas, CA, USA). ALP activity was measured using commercial kits from MyBioSource (Cat. No. MBS165203).

#### 4.5.3. Lipid Profile Analysis

Serum total cholesterol was measured using a rat-specific QuickDetect™ ELISA kit (BioVision, USA; Cat. No. K4436-100) at 450 nm. Triglycerides (TG) were determined using the Spectrum Diagnostics GPO-PAP colorimetric assay (REF: 314 001–314 010) at 546 nm. HDL-C was measured after precipitation of LDL and VLDL fractions using the Spectrum Diagnostics HDL-C reagent (REF: 266 001/266 002), whereas LDL-C was directly quantified using the Spectrum Diagnostics enzymatic colorimetric assay (REF: 280 001/280 002) at 600 nm. All measurements were performed in duplicate according to the manufacturers’ instructions.

#### 4.5.4. Assessment of Oxidative and Antioxidant Status

Hepatic oxidative stress and antioxidant capacity were evaluated by measuring MDA, NO, GSH, SOD, and CAT in liver homogenates. Lipid peroxidation was quantified as MDA using the TBARS method [48]. Nitric oxide metabolites were determined via the Griess reaction [49]. GSH levels were assessed using the Elman-based assay [50]. Antioxidant enzyme activities were measured by standard spectrophotometric methods: SOD activity based on the inhibition of nitroblue tetrazolium reduction [51]; CAT activity was measured by monitoring hydrogen peroxide degradation [52]; and GPx activity was assessed using commercial ELISA kits from MyBioSource (Cat. No. MBS744364).

In addition, hepatic Keap1 and Nrf2 levels were determined using rat-specific ELISA kits as described in the “Protein Biomarker Determination by ELISA” subsection.

#### 4.5.5. Evaluation of Inflammatory Markers

Hepatic NF-κB, TNF-α, and IL-6 levels were quantified in liver tissue homogenates using rat-specific ELISA kits as described in the “Protein Determinations by ELISA” subsection.

#### 4.5.6. Evaluation of Apoptotic Markers

Hepatic cytochrome c, Bcl-2, and caspase-3 levels were measured in liver tissue homogenates using rat-specific ELISA kits as described above.

#### 4.5.7. Gene Expression Analysis

Quantitative reverse transcription polymerase chain reaction (RT-qPCR) was performed to investigate hepatic transcriptional alterations associated with lipid metabolism and mitochondrial homeostasis. Total RNA was isolated from liver tissues and reverse-transcribed into complementary DNA (cDNA). The relative mRNA expression levels of genes involved in de novo lipogenesis, including fatty acid synthase (*Fasn*) and sterol regulatory element-binding protein 1c (*SREBP-1c*), as well as fatty-acid oxidation and metabolic regulation markers, including peroxisome proliferator-activated receptor alpha (*Ppara*/*PPAR-α*), AMP-activated protein kinase (*AMPK*), and peroxisome proliferator-activated receptor gamma coactivator 1-alpha (*PGC-1α*), were evaluated. Mitochondrial dynamics were further assessed by determining the hepatic mRNA expression of mitofusin 2 (*Mfn2*). Glyceraldehyde-3-phosphate dehydrogenase (*GAPDH*) was used as the internal reference gene for normalization. Relative gene expressions were calculated using the $2^{-\Delta \Delta Ct}$ method. The primer sequences used for RT-qPCR analysis are presented in Table 1.

### 4.6. Histopathological Examination

Liver samples were fixed, paraffin-embedded, and stained with hematoxylin and eosin (H&E) to assess overall hepatic architecture, including hepatocyte integrity, vascular changes, and degenerative or necrotic lesions. Histological evaluation focused on identifying high-fat diet-induced alterations and determining the extent of structural improvement following treatment [10,53].

### 4.7. Immunohistochemical Assessment of Nrf2

Immunohistochemistry was performed to evaluate the hepatic expression of nuclear factor erythroid 2-related factor 2 (Nrf2) (CAT # NBP3-13682; Novus Biologicals, Littleton, CO, USA), a key regulator of cellular antioxidant defense and metabolic homeostasis. Paraffin-embedded liver sections were deparaffinized, rehydrated, and subjected to antigen retrieval, followed by incubation with primary antibodies specific for Nrf2. Detection was achieved using HRP-labeled secondary antibodies and DAB chromogen, producing a brown cytoplasmic and/or nuclear signal corresponding to positive immunoreactivity. Stained sections were counterstained with hematoxylin and examined microscopically to assess the localization and intensity of marker expression. Semi-quantitative scoring was used to compare expression levels across experimental groups, enabling evaluation of treatment-induced modulation of oxidative stress and metabolic regulatory pathways. Immunoreactive site visualization was done using DAB as the chromogenic substrate. Lastly, sections were counterstained using hematoxylin and mounted on microscope slides for bright-field illumination. Semi-quantitative analysis of immunohistochemical staining was performed using ImageJ Fiji software (version 1.52n), with hematoxylin and DAB (H-DAB) image deconvolution, and subsequent quantitative analysis was performed to evaluate staining intensity and distribution. The percentage area of Nrf-2 immunoreactivity was quantified at a magnification of 400× in all experimental groups [54].

### 4.8. Statistical Analysis

Before applying parametric statistical analysis, all datasets were tested for normality and homogeneity of variance. Normality was assessed using the Shapiro–Wilk test, while homogeneity of variance was evaluated using Levene’s test. All measured parameters fulfilled the assumptions of normal distribution and variance homogeneity; therefore, one-way ANOVA followed by Tukey’s multiple-comparison post hoc test was applied. Tukey’s test was used to control multiple pairwise comparisons among experimental groups within each measured biomarker. Differences were considered statistically significant at *p* < 0.05.

Percentage change relative to the control group was calculated as:

$$\%\;\mathrm{of}\;\mathrm{Difference}=\frac{\mathrm{Treated}\;\mathrm{value}\;-\;\mathrm{control}\;\mathrm{value}}{\mathrm{Control}\;\mathrm{value}}\times 100$$

## 5. Conclusions

In conclusion, EGCG-functionalized selenium nanoparticles showed greater protective effects against high-fat diet-induced hepatic lipotoxicity than free EGCG, sodium selenite, and Lipanthyl under the present experimental conditions. EGCG-SeNPs improved dyslipidemia, liver injury biomarkers, oxidative stress, inflammatory responses, and mitochondrial apoptotic markers. These effects were associated with modulation of the Keap1/Nrf2 antioxidant axis, suppression of NF-κB-related inflammatory markers, regulation of lipogenic and fatty-acid oxidation-related genes, and restoration of AMPK/SIRT1/PGC-1α/MFN2-associated mRNA expression. Histopathological and immunohistochemical findings further supported the biochemical and molecular results. However, because AMPK/SIRT1/PGC-1α/MFN2 markers were assessed at the mRNA level, these findings should be interpreted as transcriptional modulation rather than definitive evidence of pathway activation at the protein or phosphorylation level. Further studies using protein expression, phosphorylation assays, and pathway inhibition approaches are required to confirm this mechanism.

## Figures and Tables

**Table 1:** Primer sequences of genes analyzed by RT-qPCR.

	Forward Sequence	Reverse Sequence
FASN	AAAGAAGCCCATCTCCCG	GCTCCACGAACTCAAACACC
SREBP-1c	CCCTGGTCTACCATAAGCTGC-3	CTTCACTCTCAATGCAGCCG
PPAR-α	TTCGACTCAAGCTGGTGTATG	GTGTGACATCCCGACAGAAA
*Mfn2*	GTG GAA TAC GCC AGT GAG AAG C	CAA CTT GCT GGC ACA GAT GAG C
*PGC-1α*	TGGATTGAAGTGGTGTAG	GTCAGTGCATCAAATGAG
*AMPK*	AAACCCACAGAAATCCAAAC	TGCTTGATTGCTCTACAAAC
*SIRT1*	GAAACCCTCAATTTCTGTTCTGCT	TTGAGATCTGCCCAGGTGGTA
*GAPDH*	ATGGTGAAGGTCGGTGTGAACG	TGGTGAAGACGCCAGTAGACTC

## Data Availability

The original contributions presented in this study are included in the article. Further inquiries can be directed to the corresponding authors.

## Abbreviations

The following abbreviations are used in this manuscript:

Abbreviation	Full Term
ALP	Alkaline phosphatase
ALT	Alanine aminotransferase
AMPK	AMP-activated protein kinase
ANOVA	Analysis of variance
ARE	Antioxidant response element
AST	Aspartate aminotransferase
Bcl-2	B-cell lymphoma 2
BUC	Badr University in Cairo
CAT	Catalase
CK-MB	Creatine kinase-MB
CN	Control group
cTnI	Cardiac troponin I
Cyt c	Cytochrome c
DAB	3,3′-Diaminobenzidine
DLS	Dynamic light scattering
EGCG	Epigallocatechin gallate
EGCG-SeNPs	Epigallocatechin gallate-functionalized selenium nanoparticles
ELISA	Enzyme-linked immunosorbent assay
FASN	Fatty acid synthase
FWHM	Full width at half maximum
GAPDH	Glyceraldehyde-3-phosphate dehydrogenase
GPx	Glutathione peroxidase
GSH	Reduced glutathione
H&E	Hematoxylin and eosin
HDL-C	High-density lipoprotein cholesterol
HFD	High-fat diet
HFD/EGCG	High-fat diet group treated with epigallocatechin gallate
HFD/EGCG-SeNPs	High-fat diet group treated with epigallocatechin gallate-functionalized selenium nanoparticles
HFD/LPL	High-fat diet group treated with Lipanthyl
HFD/Na_2_SeO_3_	High-fat diet group treated with sodium selenite
IHC	Immunohistochemistry
IL-6	Interleukin-6
Keap1	Kelch-like ECH-associated protein 1
LDL-C	Low-density lipoprotein cholesterol
LPL	Lipanthyl
MDA	Malondialdehyde
MFN2	Mitofusin 2
mRNA	Messenger ribonucleic acid
Na_2_SeO_3_	Sodium selenite
NF-κB	Nuclear factor kappa B
NO	Nitric oxide
Nrf2	Nuclear factor erythroid 2-related factor 2
PBS	Phosphate-buffered saline
PGC-1α	Peroxisome proliferator-activated receptor gamma coactivator 1-alpha
PPAR-α	Peroxisome proliferator-activated receptor alpha
ROS	Reactive oxygen species
RT-qPCR	Reverse transcription quantitative polymerase chain reaction
SeNPs	Selenium nanoparticles
SEM	Standard error of the mean
SIRT1	Sirtuin 1
SOD	Superoxide dismutase
SREBP-1c	Sterol regulatory element-binding protein 1c
TC	Total cholesterol
TEM	Transmission electron microscopy
TG	Triglycerides
TNF-α	Tumor necrosis factor-alpha
TrxR	Thioredoxin reductase
UV–Vis	Ultraviolet–visible spectroscopy
XRD	X-ray diffraction
